# Characterizing the clinical relevance of digital phenotyping data quality with applications to a cohort with schizophrenia

**DOI:** 10.1038/s41746-018-0022-8

**Published:** 2018-04-06

**Authors:** John Torous, Patrick Staples, Ian Barnett, Luis R. Sandoval, Matcheri Keshavan, Jukka-Pekka Onnela

**Affiliations:** 1 Department of Psychiatry, Beth Israel Deaconess Medical Center, Boston, MA USA; 2 Harvard Medical School, Boston, MA USA; 3 Department of Biostatistics, Harvard T.H. Chan School of Public Health, Boston, MA USA; 4 Department of Biostatistics, University of Pennsylvania, Philadelphia, PA USA

**Keywords:** Scientific data, Statistics, Software, Human behaviour, Signs and symptoms

## Abstract

Digital phenotyping, or the moment-by-moment quantification of the individual-level human phenotype in situ using data from personal digital devices and smartphones, in particular, holds great potential for behavioral monitoring of patients. However, realizing the potential of digital phenotyping requires understanding of the smartphone as a scientific data collection tool. In this pilot study, we detail a procedure for estimating data quality for phone sensor samples and model the relationship between data quality and future symptom-related survey responses in a cohort with schizophrenia. We find that measures of empirical coverage of collected accelerometer and GPS data, as well as survey timing and survey completion metrics, are significantly associated with future survey scores for a variety of symptom domains. We also find evidence that specific measures of data quality are indicative of domain-specific future survey outcomes. These results suggest that for smartphone-based digital phenotyping, metadata is not independent of patient-reported survey scores, and is therefore potentially useful in predicting future clinical outcomes. This work raises important questions and considerations for future studies; we explore and discuss some of these implications.

## Digital phenotyping: assessing data quality in schizophrenia

A pilot study shows that smartphone-collected data from patients with schizophrenia could be used to infer their mental-health status. Using smartphones as scientific data gathering tools holds great promise for understanding some of the behavioral features of psychiatric disorders and could provide an early indication of worsening symptoms. However, few studies have assessed the quality of the collected data, and thus the accuracy of clinical outcome prediction. Patrick Staples at the Harvard T. H. Chan School of Public Health in Boston, MA, and colleagues examined the relationship between data quality and future symptom-related survey responses in 16 patients with schizophrenia. They found that smartphone sensor data as well as phone-use metrics related to the completion of symptom-related surveys were significantly associated with survey results, highlighting the clinical relevance of this approach.

## Introduction

The complexity and heterogeneity of mental disorders, especially schizophrenia, has challenged psychiatry since the inception of the field.^1^ For centuries, physicians recognized that psychotic disorders were complex multifactorial diseases influenced by both biological and environmental factors. But despite recognition of this complexity and heterogeneity, quantifying mental disorders remains a challenge today.^2,3^ Advances in genetics, such as genome-wide association studies (GWAS), and in neuroimaging, such as functional MRI (fMRI), offer new tools that psychiatry has embraced to advance understanding of the genetic and neural basis of psychiatric disorders.^4^ Even more recently, smartphones and wearable sensors have been proposed as another set of tools for advancing understanding of physiological and behavioral perspectives of these disorders over time.^5^ Digital phenotyping, or the moment-by-moment quantification of the individual-level human phenotype in situ using data from smartphones and other personal digital devices, holds considerable potential for psychiatry and the collection of phone-mediated social and behavioral markers may offer a new target for biological psychiatry.^6^ However, realizing the potential of digital phenotyping requires scientific understanding of the smartphone as a scientific data gathering tool.

While there has recently been much excitement about the applications of smartphones for psychiatry, there has been markedly less focus on properties of the data measurements, or metadata. For example, data gathered from either GWAS or fMRI studies are not perfect representations of the subjects’ underlying genomes or brain activity, and ignoring the assumptions and limitations of these tools and the data they generate can lead to false interpretations, or as one aptly titled paper notes, “puzzlingly high correlations in fMRI studies of emotion, personality, and social cognition”.^7^ While there is a growing literature on the use of personal digital devices in psychiatry, few studies have verified data quality in digital phenotyping, especially in schizophrenia.^8^

Understanding the quality and properties of smartphone data is important for its proper interpretation.^9^ As an example, consider a smartphone study that monitors GPS in order to determine if there is an association between distance traveled and worsening symptoms. It is not possible to sample GPS continuously, as this would drain any phone’s battery in a few hours. Instead, apps might ping the GPS sensor at intervals with a specified frequency and duration. For example, an app might record GPS readings for 60 s once per hour. If even less data is obtained than expected from a subject, that may reflect (1) the subject turning off GPS, (2) the phone’s GPS sensor only responding to a subset of the queries for location data, (3) the GPS sampling for a duration less than requested, or (4) the GPS sampling at a different frequency than specified. If two subjects have different smartphone models or manufacturers, the GPS sensing and sensor data may differ between them. It is therefore important to understand how data is collected, not only for accurate interpretation of results but also for enhancing reproducibility of research. Considering the smartphone as a scientific measurement tool for psychiatric research, there is much we do not know at present about the reliability, sensitivity, and specificity of numerous sensors, such as GPS, accelerometer, and the microphone.

Another critical aspect of smartphone-based digital phenotyping is that the metadata generated as part of data collection is potentially clinically valuable. For example, when a smartphone pings a patient to take a symptom survey, the exact time the survey was offered, when it was started and completed can be recorded. Other data, such as time taken to answer each question and possible adjustments to previous answers, can also be recorded. This metadata may potentially contain clinically relevant information related to cognition such as attention, processing speed, and working memory. Survey completion rates that are implausibly fast or unrealistically slow may also potentially inform about the validity and quality of survey responses, which might serve as an early indication of worsening symptoms.

In this pilot study, we detail a procedure for estimating data quality for smartphone sensor data that is scheduled to be sampled according to a fixed schedule specified by investigators, and we explore clinical implications of data quality for a digital phenotyping study in a schizophrenic cohort. We display how these and other passive measures vary across subjects and time, and we model the relationship between data quality and future survey responses to questions relating to various domains of schizophrenia.

## Results

Sixteen outpatients in a state mental health clinic in active treatment with confirmed diagnosis of schizophrenia used the Beiwe smartphone app,^10^ the front-end of the Beiwe smartphone-based digital phenotyping platform, for a duration of up to 3 months. The study protocol is explained in detail in ref. ^10^ and we briefly review the clinical protocol here. Patients installed the Beiwe smartphone app onto their personal Android or iOS smartphones. The app collected two categories of data, active and passive. The active data collected in this study encompassed symptom surveys. A variety of surveys queried a total of 23 questions related to mood, anxiety, sleep, psychosis, and medication adherence, and the app prompted subjects to take the surveys at 10a.m. on every Monday, Wednesday, and Friday. Additionally, the app also collected passive data from GPS, accelerometer, anonymized call and text logs, screen on/off status, and phone battery charging status. To conserve battery, accelerometer and GPS data was collected using an alternating on-cycle–off-cycle schedule. Accelerometer data was gathered with a frequency of 10 Hz for 60 s (on-cycle), followed by 60 s of no data collection (off-cycle). GPS was scheduled to collect data with 1 Hz frequency for 60 s on-cycle, followed by 600 s off-cycle. Over the 90-day study period that subjects used the Beiwe app, they visited the clinic every 30 days to complete in-person clinical assessments of their mood, anxiety, sleep, psychotic symptoms, and general functioning. Of the 16 subjects, 5 experienced a relapse as defined by hospitalization for psychiatric reason, or an increase in the level of care or medications related to psychiatric symptoms. As stated above, we focus on the quality of passively collected data and its relationship to outcomes of interest as measured by actively collected survey data. We have focused on using accelerometer data to estimate sleep metrics from this patient cohort elsewhere.^11^

**Fig. 1:**
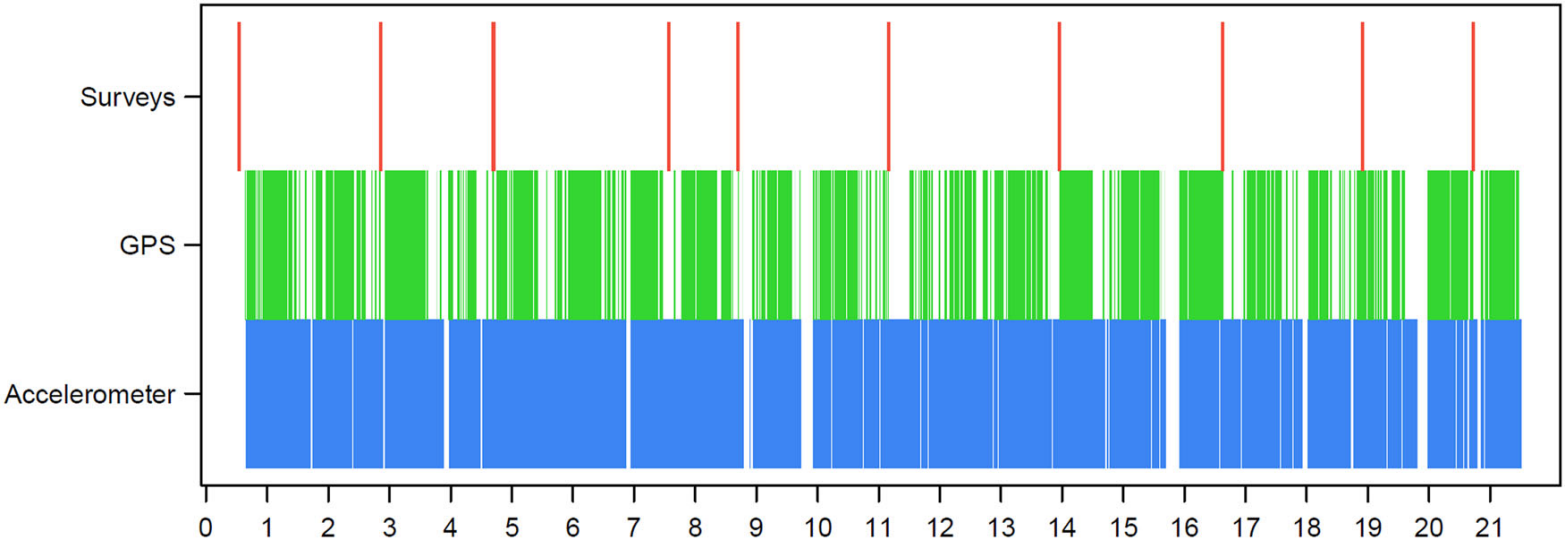
A sample of passive and active data gathered for one patient over 3 consecutive weeks. The *x*-axis shows the number of days since enrollment in continuous time, and the *y*-axis shows a sample of passive and active data gathered throughout the study

The coverage of passive data (defined in “Methods”) differs by each subject and the time since enrollment within the study. Figure 1 displays accelerometer, GPS, and survey data gathered for the first 3 weeks for one subject.

**Fig. 2:**
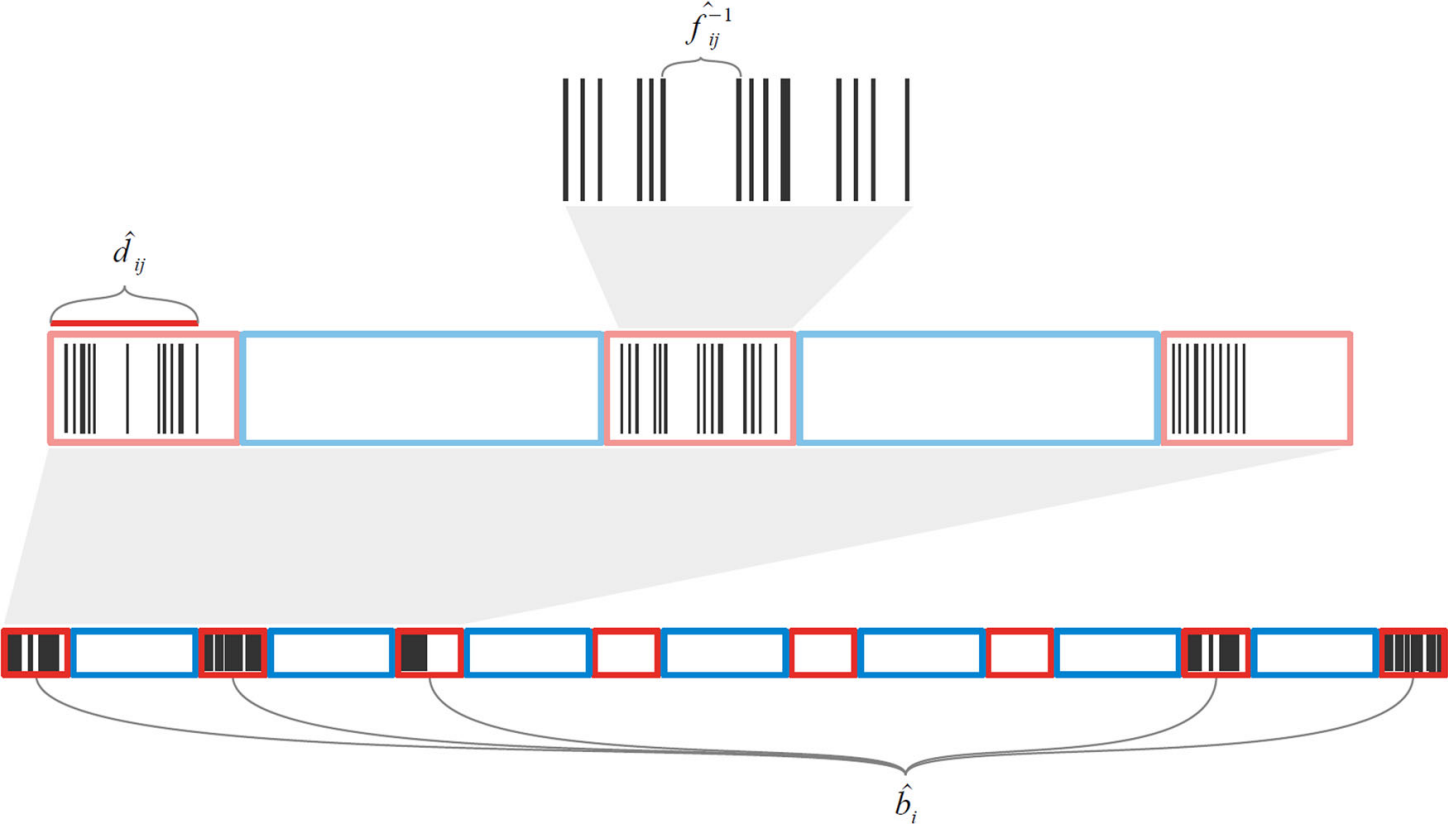
A schematic of data collection for continuous sensor data such as GPS and accelerometer. Data is assumed to be collected in a periodic fashion: red boxes show contiguous segments of time during which data is collected at a prespecified target frequency (on-cycles), and blue boxes show periods with no data collection (off-cycles). This diagram displays example data, or pings, as black lines. For day *i*, $\hat b_i$ is the estimated number of bursts, $\hat d_{ij}$ is the estimated duration of burst $j$, and $\hat f_{ij}$ is the estimated frequency of burst *j*. The estimation process for each of these quantities is given in Algorithm 1

To estimate measures of data quality we define a *burst* as a period of on-cycle time, during which data is expected to be gathered according to a fixed sampling schedule. We call a specific data measurement or observation within a burst a *ping*. Our measures of data quality include daily number of bursts and duration of each burst, as well as the frequency of pings in units of Hertz within each burst. These quantities are shown schematically in Fig. 2. In “Methods”, we define the notation and algorithm we use for estimating these quantities.

**Fig. 3:**
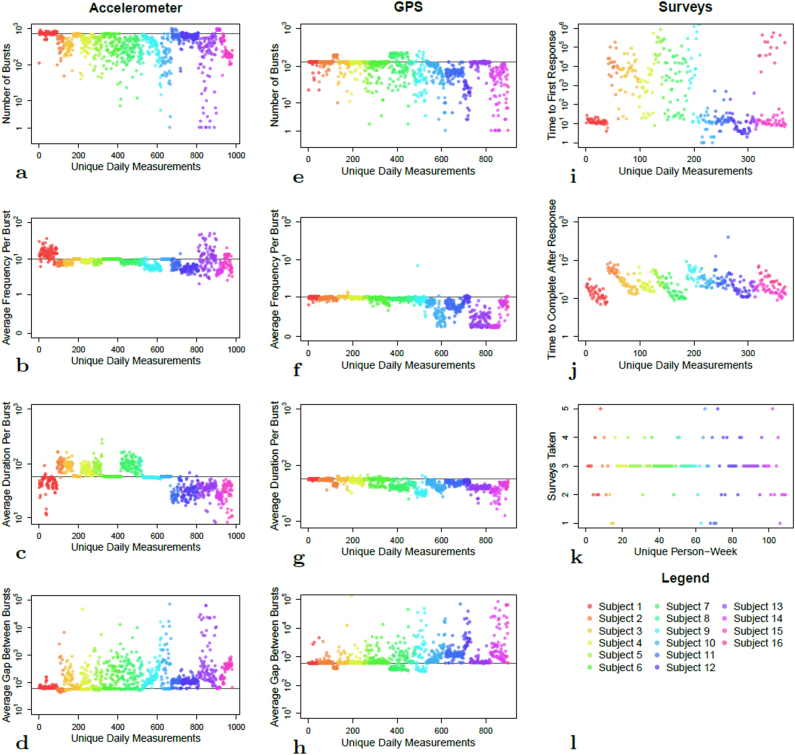
Metrics of data quality for the dataset. Within each panel, the *x*-axis shows each unique patient-day sorted and grouped by patient, the *y*-axis shows a specific metric of data quality. Colors represent different individuals, with a legend in the bottom right panel. Within each panel, patients are arranged by descending mean total data coverage, defined in “Methods”. For **a**–**d** (first column, or accelerometer) and **e**–**h** (second column, or GPS), each panel from top to bottom gives the estimated number of bursts per day, the daily average frequency of pings per burst, the daily average duration per burst (in seconds), and the daily average duration or gap between bursts (in seconds), respectively. The black lines show the expected values for these measurements, which are defined in the “Methods” section. **i**–**l** (third column, or surveys) from top to bottom show the time between subjects first responding to each phone survey after receiving a prompt (in seconds), the time from first response to survey completion (in seconds), and the total number of surveys taken per day, respectively

Figure 3 shows the estimated number of bursts per day, the average frequency per burst, the average duration per burst, and the average duration between bursts for accelerometer and GPS data for our schizophrenia cohort. The panels in the leftmost column show accelerometer data, the top panel (1st row) showing the number of bursts per day. The definition of bursts given in Algorithm 1 allows for more bursts than is expected, which is also evident in this panel. The within-burst frequency of pings (2nd row) for accelerometer varies widely by patient, which may depend on the make and type of each user’s phone. The duration per burst (3rd row) is often lower than expected, also varying widely by patient. The estimated duration between bursts (4th row) appears to have an inverse relationship to the number of bursts per day. The second column in the figure shows these same measures for GPS data. We see that the number of bursts, frequency within burst, and duration of each burst are of lower coverage than for accelerometer data. This is likely because gathering GPS data requires coordination with GPS satellites, and data collection may fail if GPS is unable to locate the satellites. This could happen, for example, if the person is inside a building. GPS can also be easily disabled on the phone by the subject. In contrast, accelerometer functions as an independent sensor within the phone and does not require coordination with external devices. The third column shows the time between the arrival of a survey and the patient’s first viewing of the survey (1st row), as well as the time from beginning the survey to completing the survey (2nd row). The time to first response for each patient appears to follow a bimodal distribution, indicating that some subjects initiate their survey response almost immediately after being prompted to do so, whereas others take several minutes or hours. In contrast, the time between observing and completing a survey seems to vary far less between patients. Finally, most subjects completed most of surveys sent to their smartphones throughout the study: of the 14 subjects that completed at least one phone survey, 12/14 (86%) completed more than two of the three surveys administered per week on average.

**Fig. 4:**
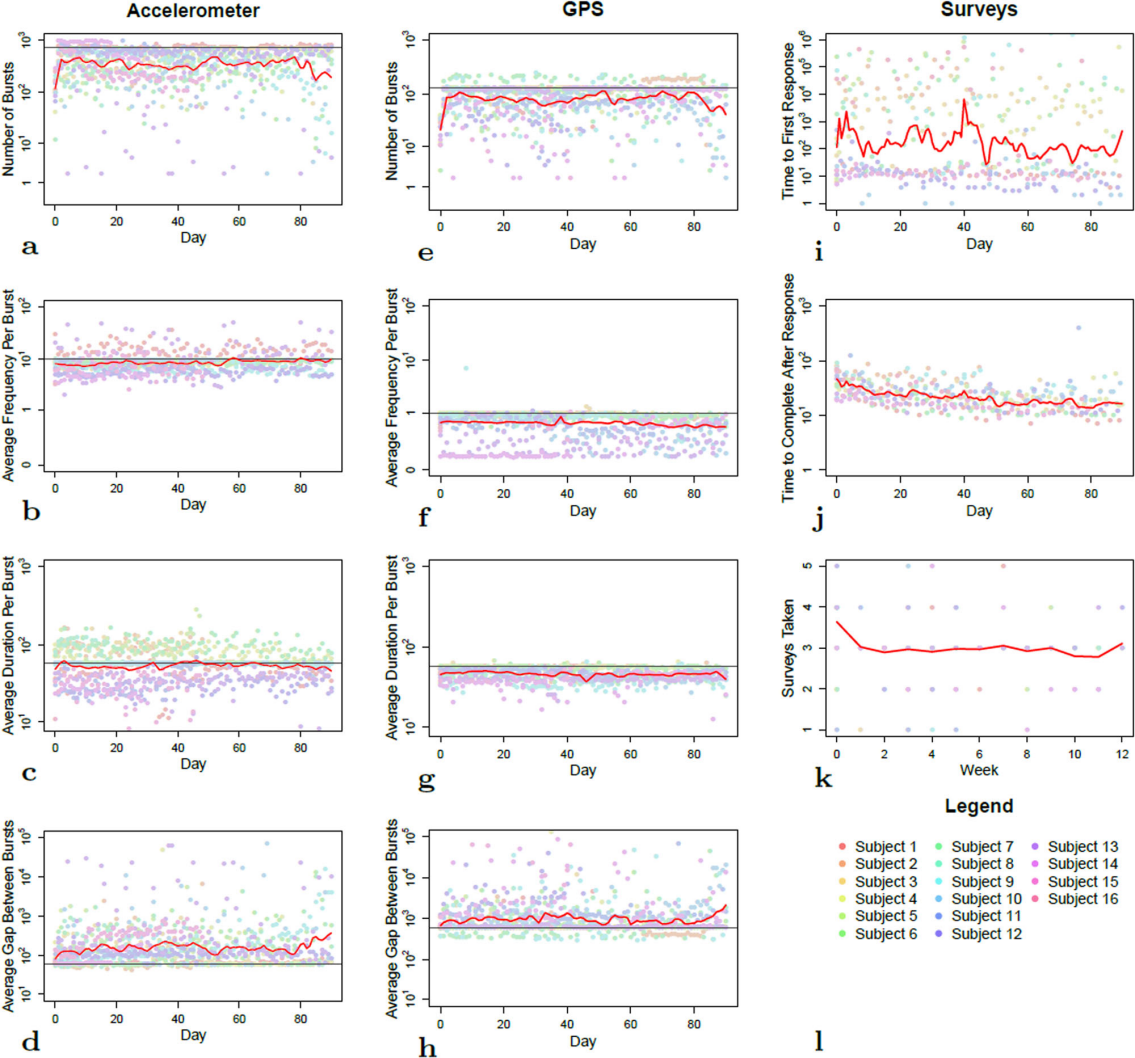
Metrics of data quality over time for the dataset. Within each panel, the *x*-axis shows the duration of time spent in the study in days for each patient, the *y*-axis shows a specific metric of data quality, sharing the same layout as Fig. 3. Colors represent different individuals, with a legend in the bottom right panel. The solid red line for each metric shows a LOWESS regression over time

Figure 4 shows these same measures of data quality over patient time, where Day 1 marks the beginning of data gathering for each individual patient. These plots show that within this cohort, data quality generally depends little on length of follow-up, and tail behavior depends on the few individuals with the longest duration of follow-up. One exception is the time between beginning a survey and completing it, which appears to decrease significantly over time across all patients. This might reflect the subjects’ familiarity with each survey over time.

Finally, it is possible that the data quality is indicative of clinical outcomes. This possibility warrants investigation because app usage patterns may contain clinically relevant and potentially predictive information about future clinical states. To estimate the strength of these associations, we created linear mixed models with data quality measures as predictors lagged by a fixed number of weeks and clinical survey measures (survey scores) as outcomes, averaged within each week for each patient. Individual survey questions presented to patients were measured on a Likert-type scale, with the following preamble:

In the last week, have you been bothered by the following problems?

Not at all = 0, Sometimes = 1, Often = 2, Frequently = 3

Covariates included total accelerometer coverage (*A*), GPS coverage (*G*), time to survey viewing (*V*), time between survey viewing and completion (*C*), and total number of surveys completed (*T*). For subject *i* and week *j*, the average survey response (*Y*) was fit according to the regression model specified in Eq. 1:

1 $$Y_{ij} = \beta _0 + \mu _i + \beta _1A_{i,j - l} + \beta _2G_{i,j - l} + \beta _3V_{i,j - l} + \beta _4C_{i,j - l} + \beta _5T_{i,j - l} + \varepsilon _{ij}.$$

**Fig. 5:**
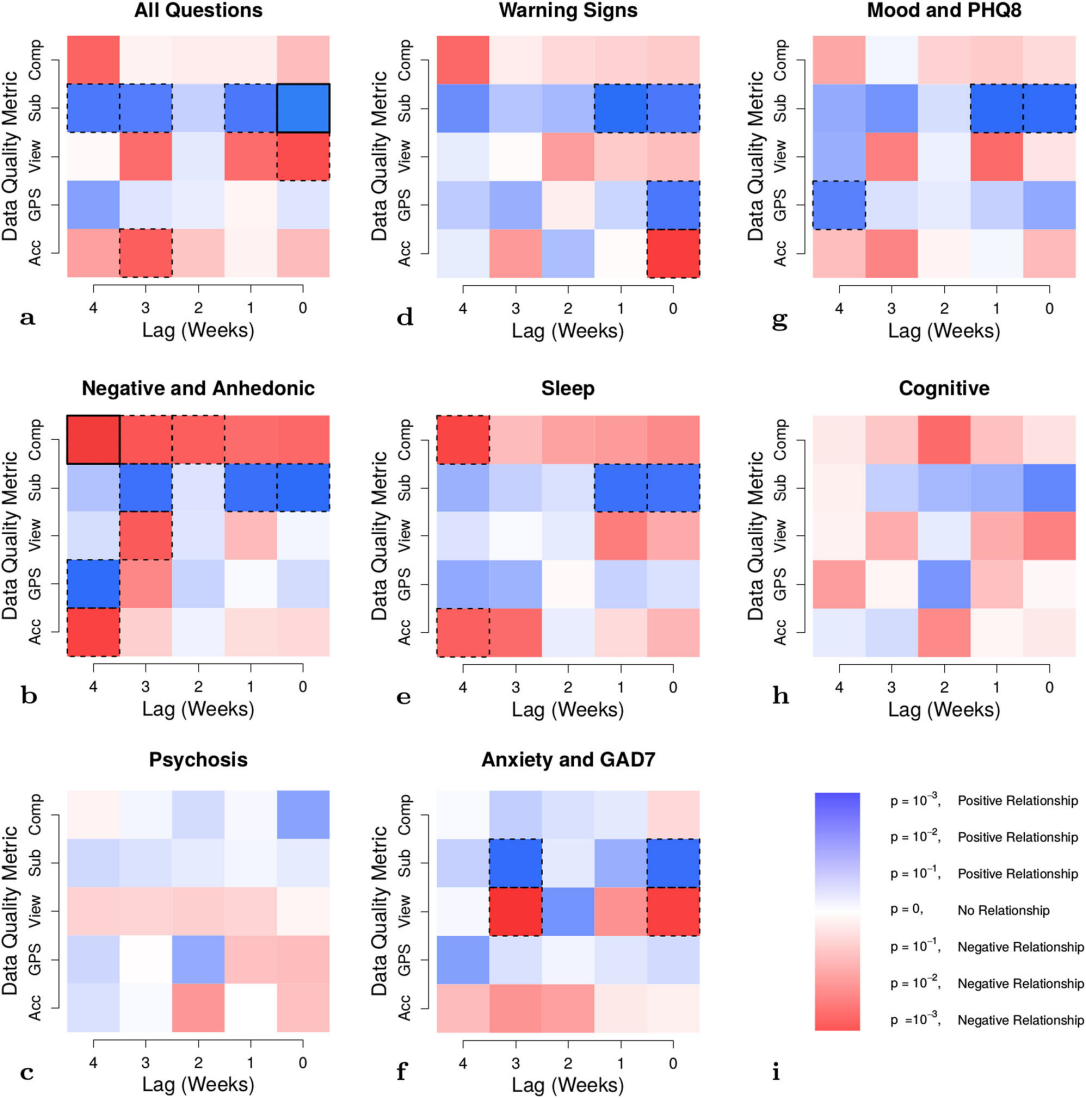
Estimates of the relationship between measures of data quality and smartphone survey domains (see Supplementary Material 1 for questions included in each domain). The *x*-axis shows the lag in weeks between the survey score (outcome) and a data quality metric (covariate). The *y*-axis shows several data quality metrics: accelerometer total coverage (Acc), GPS total coverage (GPS), the amount of time between receiving and viewing a survey (View), the time between viewing and submitting a survey (Sub), and the total number of surveys completed (Comp). Blue panels represent a positive relationship, and red panels represent a negative relationship. Color shade represents the negative log_10_ *p*-value for each model covariate. Significance without multiple correction for *p* < 0.05 is shown with a black dashed border. Correcting for multiple testing using the Benjamini–Hochberg–Yekutieli procedure, significant results are shown with a black solid border. For a numeric table of these values, see Supplementary Material 2

We generally expect higher survey scores to indicate greater effect within the domain of the survey’s subject matter, typically a negative clinical outcome. For example, when asked how often a subject “experiences little interest or pleasure in things”, we expect them to report “sometimes”, “often”, or “frequently” more frequently than “not at all” if their surveys in the past few weeks have been viewed and completed at slower rates. Survey outcomes include a subset of all questions, detailed in Supplementary Section 1. For tables of the regression coefficients, significance, and confidence intervals, see Supplementary Section 2. Figure 5 shows *p*-values and the estimated valence of each covariate. We report significance both with and without correction for multiple testing. For a comparison of the expected and observed number of significant *p*-values across all survey domains, see Supplementary Section 3. To correct for multiple testing, we employed the Benjamini–Hochberg–Yekutieli procedure,^12^ which controls the false discovery rate at a user-specified level; we chose a false discovery date of 0.05.

Across a broad range of survey domains, we find that decreased accelerometer data coverage and increased GPS data coverage are weakly associated to higher scores (see Supplementary Section 2), indicating negative clinical outcomes. Faster viewing times and slower completion rates are also associated with higher (worse) scores, particularly in questions probing anxiety. Finally, lower rates of survey completion are associated with higher (worse) survey scores, particularly for negative indicators for schizophrenia and questions probing anhedonia. We did not find statistically significant relationships between questions related to cognition or psychosis and any of these features. We find broadly similar associative patterns between survey scores and passive data lagged up to 4 weeks in the past, suggesting a stable temporal relationship between these features of data quality and survey responses.

It is possible that these relationships are confounded by technological features, such as patients’ smartphone operating system. We investigated whether the measures of metadata defined in this paper differ by Android or iOS (see Supplementary Section 4). We find that data from patients with Android phones exhibits significantly less GPS coverage recorded compared to data from patients with iOS phones, yet accelerometer data coverage did not significantly differ. Survey timings differed between users of the two operating systems as well: Android users were more prompt in beginning and completing their surveys. Given the small number of patients in this study, we cannot make strong conclusions from these differences, except to note that phone usage and measures of metadata do differ, and should be taken into account in future work.

Although we find substantial relationships between metadata and EMA surveys across a broad range of survey domains, we are also careful to note that the associations we report here are not clinical claims but rather hypothesis generating questions. To illustrate, for one participant who experienced a psychotic relapse around week 8 of the study, the metadata may hold clinical clues that the participant’s condition was changing. Unlike this patient’s initial adherence to smartphone surveys, they ceased responding to any surveys in the 3 weeks prior to relapse despite nearly complete passive data coverage as recorded by GPS and accelerometer. Although making clinical claims regarding this data will require larger clinical studies, understanding the validity and reliability of these tools and data they produce is an important first step.

## Discussion

In this study of 16 subjects with schizophrenia using the Beiwe platform for up to 90 days, we have demonstrated that the total coverage of passive data is moderately less than expected, that survey timing metrics vary greatly between subjects, and that total coverage and latency metrics are associated with self-reported clinical symptoms. These results suggest that passive data is more complex and nuanced than often assumed, and they underscore the importance of data quality in interpreting results. They also suggest that app usage patterns, irrespective of particular assessments or tasks, may themselves contain clinically relevant and potentially predictive information about future states in schizophrenia and similar disorders. To encourage open science and replication of these results, our team has released the Beiwe application into the public domain so others can replicate the results reported in this pilot study.

The mean coverage of GPS and accelerometer data for patients within the first month of follow-up was 50.2 and 46.9%, respectively. While these particular numbers are lower than ideal, they nevertheless allow for a high-resolution view of patient behavior. Indeed, traditional approaches to learning about patient mobility and movement have relied exclusively on self-reported accounts, mostly surveys, taken weeks or months apart. Smartphone-based digital phenotyping, in contrast, allows one to make a large number of daily objective measurements of these behaviors. In concrete terms, rather than observing patient location and mobility at, say, 100 bursts throughout the day, using the numbers from this particular study, we might observe their location and mobility at, say, 50 bursts. We stress that the relevant comparison is with 0 bursts, i.e., having no objective measurements at all. For clinical purposes of understanding how sedentary or social those with schizophrenia may be, 100% data coverage may not be necessary and 50% coverage might be sufficient to provide clinical insight. Even so, just as the advent of fMRI and GWAS studies highlight the danger of false discovery rates in high-dimensional data,^13^ data quality and the non-random nature of missingness need careful consideration in digital phenotyping. New methods to better quantify and account for data quality enable advances in digital phenotyping through increasing the reliability, validity, and reproducibility of research.

While our results do not permit a full explanation of why there were differences from expected values in terms of the count and duration of bursts and frequency within bursts for GPS and accelerometer, there are some plausible explanations. For example, patients might use their phones less as symptoms worsen, and their phone may enter an inactivity mode, during which time passive sensors are not recorded. Results unadjusted for multiple correction shows a weak positive relationship between GPS coverage and future survey scores, whereas accelerometer coverage and future survey scores are estimated to exhibit a weak negative association. One possible explanation for this differential relationship might be the effect of patient smartphone use on data coverage: for example, if the phone is still, it might ignore requests to record accelerometer data. Concurrently, GPS coverage might increase because their home location is easier for the Global Positioning System to locate. The phone being still would also indicate that patients interact with their phone less, which might be related to future worsening of symptoms. This and similar hypotheses would require analysis of the relationship between measures of metadata and passive measures of behavior, an important topic that falls beyond the scope of this paper.

One potential confounder is the smartphone operating system (typically Android and iOS), and model, which may react differently to apps that record and upload large quantities of data. While it is possible to minimize such differences in a study where all subjects are given the same study phone, this is impossible to do at large population scales. Furthermore, this approach ignores the reality that people use study phones differently from their own personal phones.^14^ In Supplementary Section 4, we show that on average in our sample, iPhones had less accelerometer coverage and more GPS coverage compared to Android phones. Whether type of phone itself may be a proxy for socioeconomic status or another potentially confounding variable remains an open question. Finally, the use of an app that passively measures clinically relevant behavioral data might constitute an intervention, biasing our results. For example, survey adherence in conjunction with passive monitoring might differ in survey adherence using apps that deploy phone surveys only. Understanding the nature and nuances of passive data in digital phenotyping will be an important area of research moving forward, motivating our open release of the Beiwe app in the public domain.

The relationship between survey responses, completion rates, and survey response times raises new questions about the validity of in situ surveys as measurement devices. Specifically, it is not clear whether this relationship implies that measurements of survey metadata should be treated as statistical confounders and adjusted for in the estimation of survey scores, or if the unadjusted in situ survey score should be considered the best estimator of true clinical state. Determining which of these is best for clinical use will require their comparison to other clinically relevant measures, such as relapse. We leave these analyses for future investigation.

Our results show a potential new application of passive data quality, with metrics of passive data coverage, time to first view a survey, time to complete a survey once viewed, and percentage of completed surveys, each showing a variety of temporal correlations to numerous symptoms and domains of schizophrenia. For example, our finding that those subjects reporting more severe negative symptoms and anhedonia also take longer to complete surveys on the phone and exhibit lower survey completion rates overall is intuitive, and provides a new objective measure to corroborate subjective reports. The findings that those with higher warning signs scales, suggestive of higher risk of relapse, also took longer to submit surveys supports a link between cognition, psychosis, and phone use. We speculate that completing fewer phone surveys and/or taking a longer time to open them may be an early indication or proxy for symptoms that those with schizophrenia are not immediately aware of, which accounts for the delay between the phone use data and later subject self-recognition of the deficit. However, verifying this hypothesis will require further study by both our team and others.

Like all pilot studies, ours has limitations that must be considered. First, although within the range of other smartphone mental health studies, our sample size is small. Replicating our results in more varied samples will be important future work. Since the Beiwe platform is available to researchers as open source software, and since all data collection settings of this study are captured in a Beiwe configuration file that is available from the authors, replication studies can be carried out in a relatively straightforward manner. While we minimized confounding variables that may influence unrealistic phone use, such as payments tied to use of the app, check-in calls or coaching around the app, providing subjects with new phones or study phones, and limiting inclusion criteria to either Apple or Android users, it is still possible that subjects in this study used their phones differently because they knew they were enrolled in a study that monitored their phone. This type of self-awareness would be expected to decline over time in longer studies, and one might argue that 90 days is long enough for such awareness to diminish. We also only studied a single group of patients: those in ongoing care at a state hospital, and this study does not include a healthy control group. Additionally, our sample was primarily male, and while to date there is no evidence that sex influences how those with mental illness use technology, it will be important to explore our results in the context of more diverse subject samples.

## Methods

Patients gave informed consent in writing, and methods were performed in accordance with relevant regulations and guidelines.

Let *f* be the anticipated frequency of pings within a burst, measured in pings per second. Let *d* be the expected duration of a burst, and *r* the expected rest period or gap between bursts, both measured in seconds. With *S* = 24 × 60 × 60 s within a day, the expected number of bursts per day is *b* = *S*/(*d* + *r*). The expected number of pings per day is *p* ≡ *f* × *d* × *b*.

We now specify our method to estimate the length and duration of bursts, as well as the frequency of pings within bursts. Let *D* be the number of days in the study, with indices *i* = 1,...,*D* for each day. Let *K*_*i*_ be the number of pings per day, with indices *k* = 1,...,*K*_*i*_. We write $p_i^{(k)}$ for ping *k* on day *i*, and $t(p_i^{(k)})$ for the time of ping $p_i^{(k)}$. We seek to estimate the number of bursts for each day, $\hat b_i$, with $E(\hat b_i) = b$. Let *j* be the index for each burst for day *i*, with $j = 1,...,\hat b_i$. Let $\hat p_{ij}$ and $\hat d_{ij}$ be the estimates of the number of pings for burst *j* and the duration for burst *j* on day *i*. To estimate the coverage of data for each day, we require estimates $\hat b_i$, $\hat p_{ij}$, and $\hat d_{ij}$. A visual schematic of these components is shown in Fig. 2, and our method for accomplishing this is given in Algorithm 1. In brief, we fix a duration of time **THRESHOLD**, and define a burst to be the duration of time for which the time between pings is no greater than **THRESHOLD**. When this condition is first violated, the duration and number of pings within the burst is calculated.

**Algorithm 1:**
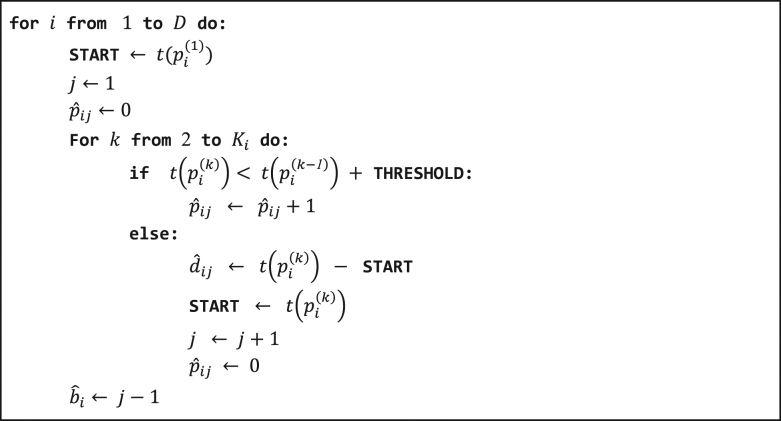
Estimation process for the duration and length of bursts, as well as the number of pings per burst

Once $\hat b_i$, $\hat p_{ij}$, and $\hat d_{ij}$ are estimated, we can define the coverage of burst count, length, and duration. Let $\hat p_i \equiv \mathop {\sum }\limits_{j = 1}^{\hat b_i} \hat p_{ij}$ be the total estimated number of pings for day *i*. The estimated average duration of bursts on day *i* is $\hat d_i \equiv \frac{1}{{\hat b_i}}\mathop {\sum }\limits_{j = 1}^{\hat b_i} \hat d_{ij}$. The estimated frequency within bursts for day $i$ is $\hat f_i \equiv \frac{{\hat p_i}}{{\hat p_i \cdot \hat d_i}}$. The total estimated coverage for day $i$ is $\hat C_i \equiv \frac{{\hat p_i}}{p} = \frac{{\hat b_i}}{b} \cdot \frac{{\hat d_i}}{d} \cdot \frac{{\hat f_i}}{{f}}$.

Values for *b*, *d*, and *f* are specified at the beginning of the study as part of the study design. In this study, Beiwe was configured to collect accelerometer and GPS data according to the following specifications. For accelerometer data, the number of bursts, within burst duration, and frequency within bursts were set to *d*_acc_ = 60, *r*_acc_ = 60, and *f*_acc_ = 10, respectively. The rule used in this paper for dividing the collected data into bursts was defined as **THRESHOLD**_acc_ = 30. For GPS, these values were set to *d*_GPS_ = 60, *r*_GPS_ = 600, and *f*_GPS_ = 1, respectively, with a rule for dividing GPS bursts defined as **THRESHOLD**_GPS_ = 30. Using these values and Algorithm 1, we estimate the coverage of data for each patient over time.

### Data availability

Data for this study will be kept on file per local IRB regulations. Although access to data in this study is restricted per study protocol^10^ due to subject identifiability, the Beiwe data collection and analysis platform are now available as open source software, affording similar external validation by research teams. In addition, the configuration files specifying the data collection schedule for subjects in this study and the R code used for analysis are made available as supplementary files to this article.

## Electronic supplementary material

Supplementary Material(DOCX 72 kb)

Code(TXT 8 kb)

## Electronic supplementary material

**Supplementary information** accompanies the paper on the *npj Digital Medicine* website (10.1038/s41746-018-0022-8).
