# Supplementary material for: Characterizing the clinical relevance of digital phenotyping data quality with applications to a cohort with schizophrenia
Source: NPJ Digit Med. 2018 Apr 6;1:15. doi: 10.1038/s41746-018-0022-8 (PMC6550248; doi:10.1038/s41746-018-0022-8)
Supplement: Supplementary file 1 — Supplementary Material(DOCX 72 kb) [file 41746_2018_22_MOESM1_ESM.docx]

**Supplementary Material**

This supplement contains details on the analysis presented in the main article. In Section 1, we detail the wording of questions asked in each survey, as well as which groupings were used for the regressions detailed in the paper. In Section 2, we provide additional details on the results for the regressions fit.

**Supplementary Section 1:
Survey Domains**

Patients took three surveys, with questions drawn from a range of clinical surveys, such as PHQ-9, GAD-8, as well as additional questions specified by the authors. We grouped the questions asked in these surveys into question domains, the outcomes of which were predicted using previous weeks’ data quality (see Figure 5 in the main article). The exact wording of each question and the questions included in each question domain are listed below.

| All Questions | Unable to cope with stress  Feeling confused or puzzled  Difficulty staying asleep  Worrying too much  Little interest or pleasure in things  Feeling suspicious  Missing doses of medications  Feeling tired  Waking up too early  Feeling nervous, scared, or anxious  Poor appetite or overeating  Trouble relaxing  Feeling bad or guilty about yourself  Hearing voices or seeing things  Feeling depressed or sad  Difficulty falling asleep  Don't feel rested after waking up  Easily annoyed or irritated  Trouble concentrating  Difficulty thinking clearly  Withdrawing from social interaction |
| --- | --- |
| Warning Signs | Unable to cope with stress  Feeling tired  Feeling depressed or sad  Feeling confused or puzzled  Don't feel rested after waking up  Feeling nervous, scared, or anxious  Little interest or pleasure in things  Trouble concentrating |
| Mood and PHQ-8 | Little interest or pleasure in things  Poor appetite or overeating  Feeling bad or guilty about yourself  Trouble concentrating  Feeling tired  Feeling depressed or sad  Trouble relaxing  Don't feel rested after waking up |
| Negative and Anhedonic | Feeling confused or puzzled  Little interest or pleasure in things  Feeling bad or guilty about yourself  Feeling depressed or sad  Trouble concentrating  Difficulty thinking clearly  Withdrawing from social interaction |
| Sleep | Difficulty falling asleep  Don't feel rested after waking up  Difficulty staying asleep  Feeling tired  Waking up too early |
| Cognitive | Trouble concentrating  Difficulty thinking clearly  Feeling confused or puzzled  Missing doses of medications |
| Psychosis | Hearing voices or seeing things  Feeling suspicious  Difficulty thinking clearly  Withdrawing from social interaction |
| Anxiety and GAD-7 | Feeling nervous, scared, or anxious  Worrying too much  Unable to cope with stress  Feeling bad or guilty about yourself  Trouble relaxing  Poor appetite or overeating |

**Supplementary Table 1:** Question domains used for prediction.

**Supplementary Section 2:
Regression Results**

In this supplementary section, we provide the estimated coefficients, their statistical significance, and their confidence intervals for the regressions specified in the main article. Each cell in the tables consists of two rows, where the first is the estimated coefficient augmented with symbols indicating significance level, and the second row is the 95% confidence interval. A legend of confidence level symbols is given directly before the tables. Results are presented in the same order as the panels in Figure 5 of the main article.

Legend: “+”: p<0.1, “*”: p<0.05, “**”: p<0.01, “***”: p<0.001

| All Questions | Lagged 4 Weeks | Lagged 3 Weeks | Lagged 2 Weeks | Lagged 1 Week | No Lag |
| --- | --- | --- | --- | --- | --- |
| (Intercept) | -0.004*  (-0.007, 0) | 0  (-0.005, 0.004) | 0.001  (-0.004, 0.007) | -0.001  (-0.005, 0.004) | -0.002  (-0.006, 0.003) |
| GPS Total Coverage | 0.19*  (0.012, 0.368) | 0.25*  (0.004, 0.497) | 0.126  (-0.181, 0.434) | 0.25*  (0.004, 0.497) | 0.367**  (0.139, 0.595) |
| Acc Total Coverage | -0.005  (-0.069, 0.06) | -0.084+  (-0.169, 0.002) | 0.012  (-0.087, 0.111) | -0.081*  (-0.157, -0.004) | -0.083*  (-0.157, -0.009) |
| Log10 Time to Begin Survey | 0.291  (-0.132, 0.714) | 0.083  (-0.478, 0.645) | 0.103  (-0.561, 0.766) | -0.11  (-0.654, 0.434) | 0.093  (-0.349, 0.535) |
| Log10 Begin to Complete Survey | -0.21  (-0.537, 0.116) | -0.421*  (-0.831, -0.012) | -0.243  (-0.753, 0.267) | -0.032  (-0.461, 0.398) | -0.186  (-0.527, 0.155) |
| Survey Completion Rate | 0.294  (-0.159, 0.748) | 0.463+  (-0.047, 0.973) | 0.361  (-0.243, 0.966) | 0.723*  (0.148, 1.298) | 0.587*  (0.034, 1.141) |

| Warning Signs | Lagged 4 Weeks | Lagged 3 Weeks | Lagged 2 Weeks | Lagged 1 Week | No Lag |
| --- | --- | --- | --- | --- | --- |
| (Intercept) | -0.024*  (-0.048, -0.001) | -0.005  (-0.027, 0.017) | 0.002  (-0.02, 0.024) | -0.011  (-0.03, 0.008) | -0.01  (-0.03, 0.009) |
| GPS Total Coverage | 0.366+  (-0.025, 0.757) | 0.182  (-0.271, 0.635) | 0.278  (-0.151, 0.707) | 0.518**  (0.15, 0.887) | 0.383*  (0.012, 0.753) |
| Acc Total Coverage | 0.03  (-0.113, 0.173) | -0.018  (-0.172, 0.136) | -0.086  (-0.223, 0.051) | -0.061  (-0.176, 0.053) | -0.062  (-0.179, 0.056) |
| Log10 Time to Begin Survey | 0.341  (-0.511, 1.194) | 0.492  (-0.435, 1.419) | -0.037  (-0.936, 0.862) | 0.059  (-0.737, 0.855) | 0.799*  (0.118, 1.479) |
| Log10 Begin to Complete Survey | 0.097  (-0.606, 0.799) | -0.468  (-1.19, 0.255) | 0.271  (-0.432, 0.973) | 0.109  (-0.52, 0.737) | -0.797**  (-1.339, -0.255) |
| Survey Completion Rate | 0.04  (-0.729, 0.81) | 0.369  (-0.426, 1.165) | 0.361  (-0.441, 1.163) | 0.423  (-0.337, 1.183) | 0.636+  (-0.094, 1.367) |

| Mood and PHQ8 | Lagged 4 Weeks | Lagged 3 Weeks | Lagged 2 Weeks | Lagged 1 Week | No Lag |
| --- | --- | --- | --- | --- | --- |
| (Intercept) | -0.014  (-0.03, 0.003) | 0.002  (-0.016, 0.019) | -0.007  (-0.026, 0.012) | -0.006  (-0.024, 0.012) | -0.005  (-0.028, 0.017) |
| GPS Total Coverage | 0.14  (-0.173, 0.453) | 0.306  (-0.069, 0.681) | 0.16  (-0.226, 0.546) | 0.401*  (0.056, 0.747) | 0.528*  (0.1, 0.956) |
| Acc Total Coverage | 0.07  (-0.044, 0.184) | -0.129+  (-0.259, 0.001) | 0.033  (-0.09, 0.155) | -0.117*  (-0.222, -0.012) | -0.021  (-0.153, 0.11) |
| Log10 Time to Begin Survey | 0.791*  (0.107, 1.474) | 0.046  (-0.772, 0.865) | -0.079  (-0.874, 0.715) | -0.016  (-0.745, 0.713) | 0.327  (-0.445, 1.099) |
| Log10 Begin to Complete Survey | -0.452  (-1.013, 0.108) | -0.59+  (-1.206, 0.027) | -0.071  (-0.704, 0.562) | 0.152  (-0.447, 0.75) | -0.26  (-0.886, 0.366) |
| Survey Completion Rate | 0.215  (-0.422, 0.852) | 0.685+  (-0.032, 1.402) | 0.431  (-0.27, 1.131) | 0.62+  (-0.083, 1.323) | 0.225  (-0.553, 1.004) |

| Negative and Anhedonic | Lagged 4 Weeks | Lagged 3 Weeks | Lagged 2 Weeks | Lagged 1 Week | No Lag |
| --- | --- | --- | --- | --- | --- |
| (Intercept) | -0.034***  (-0.052, -0.016) | -0.008  (-0.025, 0.008) | -0.023*  (-0.043, -0.004) | -0.022+  (-0.043, 0) | -0.02+  (-0.041, 0.001) |
| GPS Total Coverage | 0.109  (-0.182, 0.399) | 0.38*  (0.027, 0.733) | 0.102  (-0.295, 0.499) | 0.439*  (0.064, 0.813) | 0.488*  (0.115, 0.862) |
| Acc Total Coverage | 0.021  (-0.085, 0.127) | -0.126*  (-0.247, -0.005) | 0.039  (-0.087, 0.165) | -0.065  (-0.18, 0.05) | 0.011  (-0.106, 0.129) |
| Log10 Time to Begin Survey | 0.929**  (0.284, 1.574) | -0.389  (-1.158, 0.38) | 0.123  (-0.706, 0.953) | -0.093  (-0.883, 0.696) | 0.159  (-0.539, 0.857) |
| Log10 Begin to Complete Survey | -0.693**  (-1.214, -0.172) | -0.304  (-0.88, 0.272) | 0.131  (-0.521, 0.783) | -0.124  (-0.765, 0.517) | -0.178  (-0.727, 0.371) |
| Survey Completion Rate | 0.415  (-0.175, 1.005) | 0.619+  (-0.049, 1.286) | 0.406  (-0.337, 1.15) | 0.645+  (-0.088, 1.379) | 0.295  (-0.445, 1.035) |

| Sleep | Lagged 4 Weeks | Lagged 3 Weeks | Lagged 2 Weeks | Lagged 1 Week | No Lag |
| --- | --- | --- | --- | --- | --- |
| (Intercept) | -0.055**  (-0.095, -0.015) | -0.033+  (-0.071, 0.005) | -0.033+  (-0.072, 0.006) | -0.025  (-0.064, 0.013) | -0.03+  (-0.065, 0.005) |
| GPS Total Coverage | 0.275  (-0.182, 0.732) | 0.126  (-0.378, 0.63) | 0.12  (-0.367, 0.607) | 0.497*  (0.057, 0.938) | 0.442*  (0.035, 0.85) |
| Acc Total Coverage | 0.043  (-0.123, 0.21) | -0.002  (-0.174, 0.17) | 0.034  (-0.119, 0.187) | -0.124+  (-0.257, 0.009) | -0.082  (-0.211, 0.047) |
| Log10 Time to Begin Survey | 0.5  (-0.455, 1.455) | 0.262  (-0.719, 1.243) | -0.206  (-1.182, 0.771) | 0.185  (-0.716, 1.086) | 0.206  (-0.561, 0.973) |
| Log10 Begin to Complete Survey | -0.879*  (-1.689, -0.07) | -0.694+  (-1.488, 0.1) | 0.163  (-0.629, 0.954) | -0.152  (-0.909, 0.604) | -0.363  (-0.969, 0.243) |
| Survey Completion Rate | 0.72  (-0.146, 1.587) | 0.817+  (-0.037, 1.671) | 0.611  (-0.242, 1.464) | 0.671  (-0.154, 1.496) | 0.835*  (0.028, 1.643) |

| Cognitive | Lagged 4 Weeks | Lagged 3 Weeks | Lagged 2 Weeks | Lagged 1 Week | No Lag |
| --- | --- | --- | --- | --- | --- |
| (Intercept) | -0.001  (-0.008, 0.006) | -0.019  (-0.066, 0.027) | -0.012  (-0.055, 0.03) | -0.015  (-0.053, 0.022) | -0.006  (-0.043, 0.03) |
| GPS Total Coverage | -0.007  (-0.063, 0.049) | 0.21  (-0.282, 0.703) | 0.219  (-0.314, 0.752) | 0.27  (-0.183, 0.724) | 0.407+  (-0.033, 0.847) |
| Acc Total Coverage | -0.002  (-0.022, 0.018) | -0.084  (-0.238, 0.069) | 0.013  (-0.169, 0.194) | -0.085  (-0.234, 0.064) | -0.117  (-0.26, 0.026) |
| Log10 Time to Begin Survey | -0.099  (-0.254, 0.056) | -0.032  (-0.961, 0.896) | 0.775  (-0.351, 1.901) | -0.447  (-1.382, 0.489) | -0.152  (-1.095, 0.791) |
| Log10 Begin to Complete Survey | 0.034  (-0.142, 0.21) | 0.241  (-0.562, 1.043) | -0.557  (-1.405, 0.292) | -0.073  (-0.789, 0.643) | -0.089  (-0.813, 0.634) |
| Survey Completion Rate | 0.436+  (-0.015, 0.886) | 0.335  (-0.461, 1.131) | 0.172  (-0.788, 1.131) | 0.803+  (-0.007, 1.613) | 0.682  (-0.185, 1.55) |

| Psychosis | Lagged 4 Weeks | Lagged 3 Weeks | Lagged 2 Weeks | Lagged 1 Week | No Lag |
| --- | --- | --- | --- | --- | --- |
| (Intercept) | 0.002  (-0.05, 0.055) | 0.014  (-0.03, 0.057) | 0.018  (-0.021, 0.057) | 0.006  (-0.036, 0.048) | 0.036*  (0, 0.071) |
| GPS Total Coverage | 0.209  (-0.265, 0.683) | 0.153  (-0.256, 0.562) | 0.068  (-0.325, 0.46) | 0.097  (-0.295, 0.488) | 0.095  (-0.258, 0.449) |
| Acc Total Coverage | -0.046  (-0.215, 0.123) | -0.038  (-0.173, 0.096) | -0.056  (-0.18, 0.069) | -0.044  (-0.16, 0.072) | -0.019  (-0.128, 0.09) |
| Log10 Time to Begin Survey | 0.219  (-0.699, 1.137) | 0.058  (-0.734, 0.85) | 0.594  (-0.243, 1.432) | -0.138  (-0.904, 0.628) | -0.185  (-0.828, 0.458) |
| Log10 Begin to Complete Survey | 0.352  (-0.447, 1.151) | 0.068  (-0.553, 0.69) | -0.368  (-1.008, 0.272) | -0.083  (-0.736, 0.57) | -0.256  (-0.771, 0.259) |
| Survey Completion Rate | -0.23  (-1.128, 0.668) | 0.068  (-0.674, 0.81) | 0.263  (-0.532, 1.057) | 0.56  (-0.128, 1.247) | 0.51  (-0.142, 1.163) |

| Anxiety and GAD7 | Lagged 4 Weeks | Lagged 3 Weeks | Lagged 2 Weeks | Lagged 1 Week | No Lag |
| --- | --- | --- | --- | --- | --- |
| (Intercept) | -0.001  (-0.025, 0.023) | 0.008  (-0.017, 0.032) | 0.016  (-0.008, 0.039) | 0.008  (-0.017, 0.034) | 0.002  (-0.029, 0.033) |
| GPS Total Coverage | 0.104  (-0.187, 0.396) | 0.391*  (0.063, 0.72) | 0.011  (-0.34, 0.362) | 0.17  (-0.169, 0.508) | 0.466*  (0.053, 0.879) |
| Acc Total Coverage | 0.006  (-0.1, 0.112) | -0.175**  (-0.289, -0.061) | 0.086  (-0.025, 0.197) | -0.086  (-0.19, 0.019) | -0.153*  (-0.283, -0.023) |
| Log10 Time to Begin Survey | 0.384  (-0.292, 1.059) | 0.14  (-0.605, 0.885) | -0.002  (-0.729, 0.725) | 0.085  (-0.641, 0.811) | 0.13  (-0.617, 0.878) |
| Log10 Begin to Complete Survey | -0.216  (-0.754, 0.321) | -0.378  (-0.925, 0.168) | -0.401  (-0.971, 0.168) | -0.112  (-0.692, 0.468) | -0.083  (-0.687, 0.521) |
| Survey Completion Rate | 0.289  (-0.385, 0.963) | 0.377  (-0.297, 1.052) | 0.348  (-0.294, 0.99) | 0.656+  (-0.038, 1.351) | 0.466  (-0.316, 1.248) |

**Supplementary Table 2:** Coefficients, significance, and confidence intervals for the regressions specified in the main article.

**Supplementary Section 3:
Observed and Expected Number of *p-*Values**

In this supplementary section, we show the observed and expected number of uncorrected *p*-values for each panel in Figure 5 and sub-tables of Supplementary Table 2. Without correcting for multiple testing, we observe the distribution of the number of *p*-values less than 0.05 across all subject domains is greater than the expected distribution. This provides evidence of a relationship between the measures of metadata described in the paper and EMA survey domains. For *p*-values corrected for multiple testing, also see Figure 5.


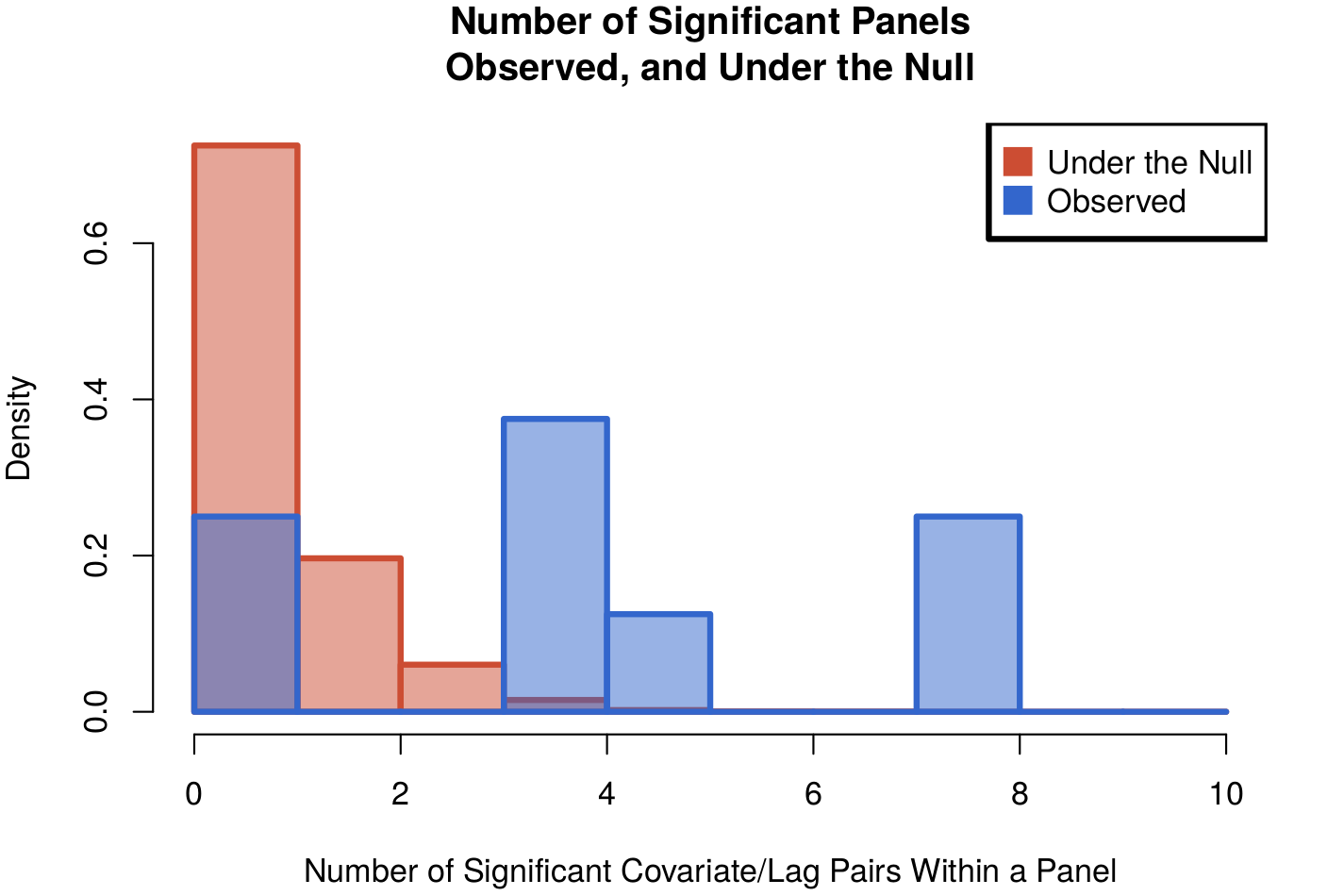


**Supplementary Figure 1:** The observed and expected *p*-values from Figure 5 and Supplementary Table 2.

**Supplementary Section 4:
Difference By Phone Type**

In this supplementary section, we provide summary statistics on data quality subdivided by phone type. The difference between these measures is given in the third row, along with 95% confidence intervals in parentheses. With the exception of accelerometer coverage, we observe a significant difference between each data quality metric by phone type.

| All Questions | Accelerometer Coverage | GPS Coverage | Time to Present | Time to Submission | n |
| --- | --- | --- | --- | --- | --- |
| iPhone | 0.473 | 0.516 | 2.724 | 1.371 | 9 |
| Android | 0.502 | 0.300 | 1.747 | 1.225 | 7 |
| Difference | -0.029 (-0.170, 0.112) | 0.217 (0.067, 0.372) | 0.977 (0.290, 1.665) | 0.146 (0.021,0.271) | 2 |

**Supplementary Table 3:** Differences in coverage by phone type. All measures significantly differ from each other, with the exception of accelerometer coverage.
